# Solar Heat

**Published:** 1855-03

**Authors:** 


Solar Heat.—G. W. Eveleth, in a communication to the National Intelligencer, broaches the following curious theory respecting solar heat:

The idea that the heat at the different planets is in proportion to the squares of their distances for the sun, is incorrect, I think. Suppose there to be but two bodies in the universal space, namely, the sun and a planet. The sun radiates heat as now. The planet is the receiver of this heat; the receiver of it all, since there can be no manifestation of heat without matter. It receives it all, whether near or distant from the sun. To be sure, the heat, then as now, is lessened in intensity, is *divided* so as to cover a greater surface—greater in proportion to the square of the distance—the further it extends outward; still, the *quantity* must be the same at all distances. This quantity *converges* to the planet as to a focus. Now, all the planets are convergers of the sun's heat, each one of them converging a quantity proportional to its size; that is, proportional to the number of particles composing it, among which for the heat to penetrate. Then Jupiter, one of the planets most distant from the sun, is receiving the greatest amount of heat; and Mercury, the nearest one to him, is receiving the least amount, (leaving the asteroids out of the account.) The oldest planets have been longest receiving heat from the sun, and have passed through the greatest number of *stages of development*, and contain the greatest amount of *internal fire*, reckoning that which has found vent through volcanoes, and which has carried with it, away into space, the matter composing the multitude of moons, revolving about these oldest planets—Jupiter, Saturn, &c.

